# Differential Impacts of Virus Diversity on Biomass Production of a Native and an Exotic Grass Host

**DOI:** 10.1371/journal.pone.0134355

**Published:** 2015-07-31

**Authors:** Erin A. Mordecai, Madeleine Hindenlang, Charles E. Mitchell

**Affiliations:** Department of Biology, University of North Carolina at Chapel Hill, Chapel Hill, North Carolina, United States of America; Cary Institute of Ecosystem Studies, UNITED STATES

## Abstract

Pathogens are common and diverse in natural communities and have been implicated in the success of host invasions. Yet few studies have experimentally measured how pathogens impact native versus exotic hosts, particularly when individual hosts are simultaneously coinfected by diverse pathogens. To estimate effects of interactions among multiple pathogens within host individuals on both transmission of pathogens and fitness consequences for hosts, we conducted a greenhouse experiment using California grassland species: the native perennial grass *Nassella* (*Stipa*) *pulchra*, the exotic annual grass *Bromus hordeaceus*, and three virus species, *Barley yellow dwarf virus-PAV*, *Barley yellow dwarf virus-MAV*, and *Cereal yellow dwarf virus-RPV*. In terms of virus transmission, the native host was less susceptible than the exotic host to MAV. Coinfection of PAV and MAV did not occur in any of the 157 co-inoculated native host plants. In the exotic host, PAV infection most strongly reduced root and shoot biomass, and coinfections that included PAV severely reduced biomass. Infection with single or multiple viruses did not affect biomass in the native host. However, in this species the most potentially pathogenic coinfections (PAV + MAV and PAV + MAV + RPV) did not occur. Together, these results suggest that interactions among multiple pathogens can have important consequences for host health, which may not be predictable from interactions between hosts and individual pathogens. This work addresses a key empirical gap in understanding the impact of multiple generalist pathogens on competing host species, with potential implications for population and community dynamics of native and exotic species. It also demonstrates how pathogens with relatively mild impacts independently can more substantially reduce host performance in coinfection.

## Introduction

Pathogens are ubiquitous in wild plant communities [1,2]. Despite the occasionally dramatic emergence and impacts of epidemic pathogens such as sudden oak death and chestnut blight [3,4], most plant pathogens are consistently present (i.e., endemic) at low levels in natural plant communities [1]. Far less is known about the consequences of these endemic pathogens for their hosts, yet they can be important for regulating plant populations and communities [5]. Though less obvious, interactions between multiple pathogen species can shape plant community composition by having subtly different impacts on different host species [6]. In plants, simultaneous infection by multiple pathogens can alter disease severity and mortality rates [7]. In this paper, we investigate the consequences of multiple interacting virus species for two plant host species that are key components of California grasslands.

Recent work has shown that complex interactions between vector and virus species shape the community composition of a group of plant viruses, the barley yellow dwarf viruses (BYDVs) [8–10]. However, we do not yet know how virus and vector interactions, and in turn the composition of the virus communities within hosts (i.e., the virus infra-community (sensu [11]), differentially affect host plants. Moreover, BYDVs are thought to contribute to annual grass dominance and native grass suppression in California grasslands [12], and although coinfection by multiple BYDVs is common in the field [8,13], the consequences of coinfection for host population and community dynamics remain unknown.

Here, we address a key empirical gap that will inform how virus diversity and composition may impact plant communities. To our knowledge, no published studies have inoculated individual wild plants with more than two virus species, and research on fungal pathogens of plants suggests that the joint effect of three pathogens may not be simply predicted from the effects of two pathogens, and may vary among host species [6]. We use a greenhouse inoculation experiment to ask how infection and coinfection with three virus species affects the individual performance (measured by root and shoot biomass) of two plant species: *Nassella* (*Stipa*) *pulchra*, a perennial bunchgrass native to California, and *Bromus hordeaceus*, an invasive annual grass now ubiquitous in much of the California grassland habitat. The virus species are three common barley yellow dwarf viruses: *Barley yellow dwarf virus-PAV*, *Barley yellow dwarf virus*-*MAV*, and *Cereal yellow dwarf virus-RPV* (hereafter, PAV, MAV, and RPV, respectively). These viruses are systemic through phloem tissue, and are obligately transmitted by aphids, chiefly two species: *Rhopalosiphum padi* transmits PAV and RPV, and *Sitobion avenae* transmits PAV and MAV.

Previous work has shown that California native perennial grasses tend to have lower susceptibility to PAV infection than exotic annual grasses [14], that PAV and MAV tend to cross-protect against each other [15,16], and that PAV infection facilitates RPV infection (Marchetto & Power, pers. comm.), but this interaction depends on nutrient availability [10]. In addition, PAV infection reduced both perennial grass and *Bromus hordeaceus* biomass, basal area, and seed production, as well as perennial grass survival in the field [17–19]. Based on these previous results, we expected that (1) the viruses would have lower inoculation success in *Nassella*, (2) PAV and MAV would each have lower inoculation success when the other virus was present in a plant, (3) PAV would increase RPV inoculation success, and (4) both *Nassella* and *Bromus* would experience biomass reductions when infected that would increase with within-host virus diversity.

## Methods

### Study system and experimental design

The primary purpose of this study was to examine the rates and impacts of infection and coinfection of different BYDV species in the California native bunchgrass, *N*. *pulchra*, and the exotic grass, *B*. *hordeaceus*. Plants were grown in 938 mL D60 Deepots (Stuewe and Sons Inc, Tangent, OR) filled with Fafard 3B potting mix. Plants were inoculated via aphids with all possible combinations of three viruses, and control plants were mock-inoculated with uninfected aphids to account for the effect of aphid feeding. As a further control, three plants of each species received no aphids. In total, the experiment contained 600 plants divided into three blocks of 200 plants each (133 *N*. *pulchra* and 67 *B*. *hordeaceus* per block). The sample sizes were doubled for *Nassella* due to lower expected inoculation success from previous work [14]. All treatments and sample sizes are listed in Table A in S1 Appendix.

Seed of both host species was hand-collected at Hopland Research and Extension Center, CA, USA. To include a representative amount of genetic variation in each host species, seed used in the experiment was selected from a pooled collection of seed from multiple wild populations, and multiple maternal families within each population. The virus isolates used were originally collected and purified by Rochow et al. [20]. They have since been propagated in greenhouse *Avena sativa* L. cv. ‘Coast Black’, undergoing approximately three transmission cycles per year. These isolates are the type specimens for the three virus species, and using them facilitates comparison of our results to the extensive published experiments on these same isolates, including both comparisons among virus species, and two-way interactions among virus species ([10], e.g., [21–23]).

### Inoculations

Plants were inoculated with viruses at the three-leaf stage. Before inoculation, the vectors acquired the virus by feeding on infected plant tissue in dishes for 48 hours. Plants were inoculated with five aphids for each viral species they received, as outlined in Table A in S1 Appendix. However, to reduce the aphid load on plants co-inoculated with multiple viruses, plants were co-inoculated with PAV and RPV using five *R*. *padi* aphids that had been exposed to both viruses (these viruses are not known to interact within shared aphids). Co-inoculation of PAV and MAV using doubly exposed aphids was not possible because these viruses strongly interfere with each other within their shared aphid vector, *S*. *avenae* [24]. The resulting inoculations used five aphids per plant (5 *R*. *padi* for *R*. *padi* controls, PAV, RPV, and PAV+RPV; 5 *S*. *avenae* for *S*. *avenae* controls, MAV) or ten aphids per plant (5 *R*. *padi* + 5 *S*. *avenae* for *R*. *padi* + *S*. *avenae* controls, PAV+MAV, MAV+RPV, and PAV+MAV+RPV). The aphids were caged onto individual plants for a 72-hour inoculation access period, and then removed using es-fenvalerate (Asana XL; DuPont) non-systemic insecticide.

### Greenhouse growth and harvesting

Pots were initially seeded with 10 seeds per pot for *Nassella* and 3 seeds per pot for *Bromus* (based on the higher expected germination fraction of *Bromus*). Two weeks prior to inoculations, pots were thinned to one plant per pot. Five weeks after inoculations, all above- and below-ground tissue was harvested from each plant. Between 0.2 and 0.3 grams (fresh weight) of above-ground plant tissue was collected for ELISA virus assays (described below). The remaining above-ground tissue was weighed fresh, then dried for at least 72 hours at 60°C and weighed dry. We estimated the additional dry mass of tissue collected for ELISAs based on the ratio of dry mass to wet mass in the main tissue samples, and added this amount to the above-ground biomass. We estimated below-ground biomass by hand-washing roots to remove all soil and debris, drying them for at least 72 hours at 60°C, and weighing them. Due to time constraints, roots were washed from a subset of plants representing each infection status category.

### Determining infection status

In order to determine the infection status of each plant, we performed double-antibody sandwich indirect ELISA using monoclonal antibodies from Agdia, Inc., Elkhart IN, USA, as detailed in previous work [14,18,19,25]. Each plant was assayed for each of the three viral species. Each sample was homogenized by grinding in extraction buffer at a 1:10 mass ratio of tissue:buffer. Infection status was determined based on comparison of optical density (OD) values [26], here determined on a BioTek ELx800 microplate optical reader. The OD of each inoculated plant was corrected for background variation from sources other than virus infection. Background variation was estimated as the average OD value of mock-inoculated plants that had received the same number and species of virus-free aphids as the inoculated plant (excluding any controls for which the OD value was one or higher, which were likely contaminated). This value was then subtracted from the OD for each inoculated plant. We assumed that samples with a corrected OD value greater than 0.3 were infected. Contamination affected the last two sets of microplates run on the last day of the assay (likely due to an equipment malfunction by the microplate washer); all 130 samples from these contaminated microplates were removed from all analyses. In total, analyses included 470 plants, which were infected with all or a subset of the viruses with which they were inoculated. Of these, we had shoot biomass data for all plants and root biomass data for 463 plants.

### Data analysis

#### Infection

We used statistical models to test how the probability of becoming infected with a particular virus when inoculated (i.e., inoculation success) depended on the plant species identity and infection status with respect to the other two viruses. For each virus species, we fit a binomial generalized linear model (GLM) with a logit link, using the formula:

Infection(0,1)~Plant(BRHO,NAPU)+Virus1status(0,1)+Virus2status(0,1)

where *Infection* is the outcome for a plant individual for a given virus, *Virus 1 status* and *Virus 2 status* represent the status of the plant with respect to the other two viruses (0 = uninfected, 1 = infected), and *Plant* is the species identity of the focal individual (BRHO = *Bromus hordeaceus*, NAPU = *Nassella pulchra*).

To compare the inoculation success of the viruses, we also fit an overall model of inoculation success as a function of plant species and virus species, irrespective of infection status with respect to the other viruses:

Infection(0,1)~Plant(BRHO,NAPU)*Virus(PAV,MAV,RPV)

Because some plants were inoculated with multiple viruses, multiple data points could come from the same plant individual. We controlled for this non-independence by fitting the model as a binomial generalized linear mixed model (GLMM) with a random effect of plant individual. We additionally tested for overdispersion, but found little evidence of it (dispersion parameter = 1.005), and therefore did not correct for it.

#### Biomass responses to infection

Although we initially intended to analyze just the data from plants in which treatments were fully successful (i.e., plants that acquired all of the viruses with which they were inoculated and only those viruses), this subset of the data had low sample size for some treatments. As a result, we performed most of our analyses on the full dataset, using observed infection status (as opposed to treatment) as a predictor variable. However, we also fit one model to only the plants in which treatments were successful. For this, and all other model structures described below, we fit one model (*m*_*0*_) to the above-ground biomass data and a second model to the below-ground biomass data.

$$m_{0}:Biomass\sim Plant\left(BRHO,NAPU\right)+Treatment\left(Notreatment,Con-5-Rp,Con-5-Sa,Con-5-Rp-5-Sa,PAV,MAV,RPV,PAV-MAV,PAV-RPV,MAV-RPV,PAV-MAV-RPV\right)$$

The four control treatments are: No treatment (received no aphids), Con-5-Rp (received 5 *R*. *padi*), Con-5-Sa (received 5 *S*. *avenae*), and Con-5-Rp-5-Sa (received 5 *R*. *padi* and 5 *S*. *avenae*).

The remaining models, fit to the full dataset with observed infection status as the predictor, were linear models with the following predictors.

$$m_{1}:Biomass\sim Plant(BRHO,NAPU)$$

$$m_{2}:Biomass\sim PAVstatus\left(0,1\right)+MAVstatus\left(0,1\right)+RPVstatus\left(0,1\right)$$

$$m_{3}:Biomass\sim Infectionclass\left(S,P,M,R,PM,PR,MR,PMR\right)$$

$$m_{4}:Biomass\sim Plant\left(BRHO,NAPU\right)*\left[PAVstatus\left(0,1\right)+MAVstatus\left(0,1\right)+RPVstatus\left(0,1\right)\right]$$

$$m_{5}:Biomass\sim Plant\left(BRHO,NAPU\right)+infectionclass\left(S,P,M,R,PM,PR,MR,PMR\right)$$

$$m_{6}:Biomass\sim Plant\left(BRHO,NAPU\right)*infectionclass\left(S,P,M,R,PM,PR,MR,PMR\right)$$

$$m_{7}:Biomass\sim Plant\left(BRHO,NAPU\right)*virusnumber\left(0,1,2,3\right)$$

$$m_{8}:Biomass\sim Plant\left(BRHO,NAPU\right)*infectionstatus\left(0,1\right)$$

These models analyzed the impact of infection on biomass of each plant species in several ways: (i) infection status with respect to each of the viruses independently (*m*_*2*_ and *m*_*4*_), (ii) infection class, treating each possible combination of the three viruses as a separate class (*m*_*3*_, *m*_*5*_, and *m*_*6*_), (iii) the number of viruses infecting a plant, assuming a linear effect of virus number (*m*_*7*_), and (iv) whether or not a plant was infected with any virus (*m*_*8*_). We used these different ways of describing infection as predictors in order to explore which provided the best model fit, penalized for model complexity, via AIC. All data analysis was performed in R version 2.15.1 [27] using the package ‘lme4’ [28].

## Results

### Infection

**Fig 1:**
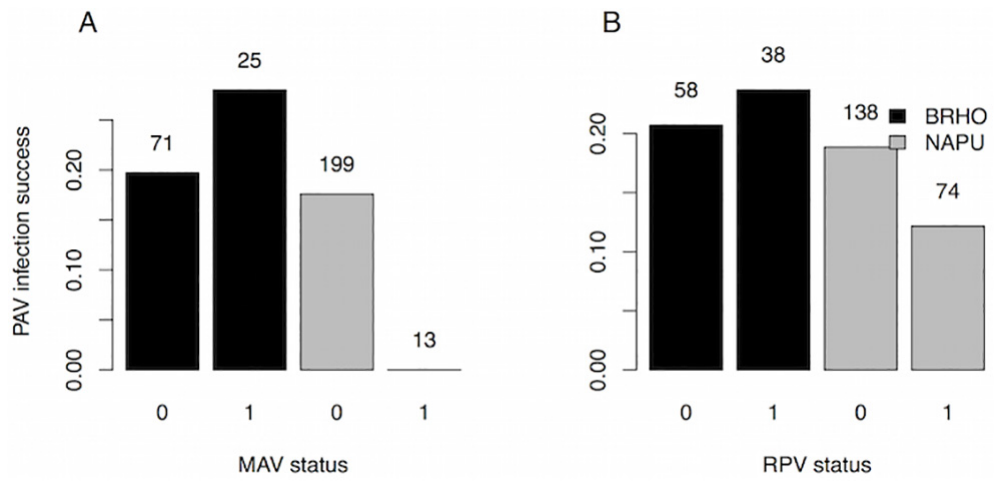
PAV inoculation success versus plant species and MAV (A) and RPV (B) infection status. Inoculation success is equal to the fraction of plants inoculated with PAV that tested positive for PAV in the ELISA. MAV status is zero for plants uninfected with MAV and one for plants infected with MAV, and likewise for RPV. Black bars are *Bromus hordeaceus* and gray bars are *Nassella pulchra*. Numbers above the bars indicate the number of plants in each group.

**Fig 2:**
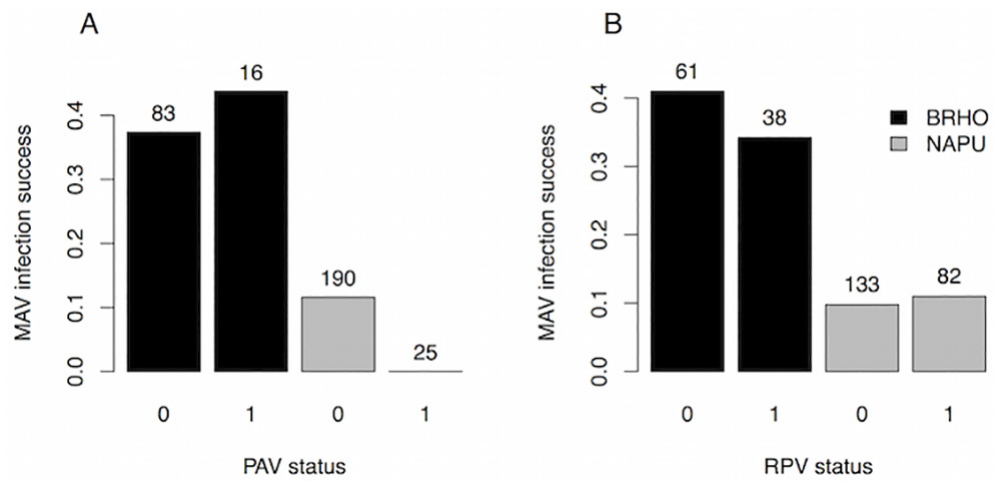
MAV inoculation success versus plant species and PAV (A) and RPV (B) infection status. Other plot features are the same as in Fig 1.

**Fig 3:**
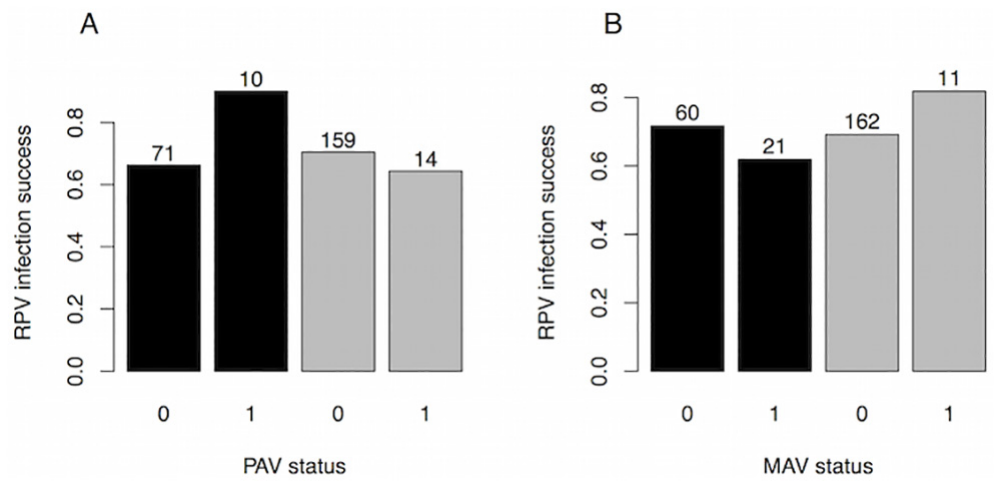
RPV inoculation success versus plant species and PAV (A) and MAV (B) infection status. Black bars represent *Bromus hordeaceus* plants, and gray bars represent *Nassella pulchra* plants. Other plot features are the same as in Fig 1.

We fit models of inoculation success for each virus separately and for all three viruses together. In the individual virus models, MAV had higher inoculation success in *Bromus* than in *Nassella* (p<0.001). Although PAV and MAV each tended to have lower inoculation success in the presence of the other, the differences were not significant (p>0.4), nor were there effects (p>0.4) of RPV infection on PAV or MAV inoculation success (Table B in S1 Appendix; Figs 1 and 2). PAV and RPV inoculation success did not significantly differ between the two plant species (p>0.2) or with virus infection status (p>0.4) (Table B in S1 Appendix; Fig 3). Notably, PAV—MAV coinfection was extremely rare: it never occurred in *Nassella* (n = 156 inoculations), and only occurred seven times in *Bromus* (n = 70 inoculations).

**Fig 4:**
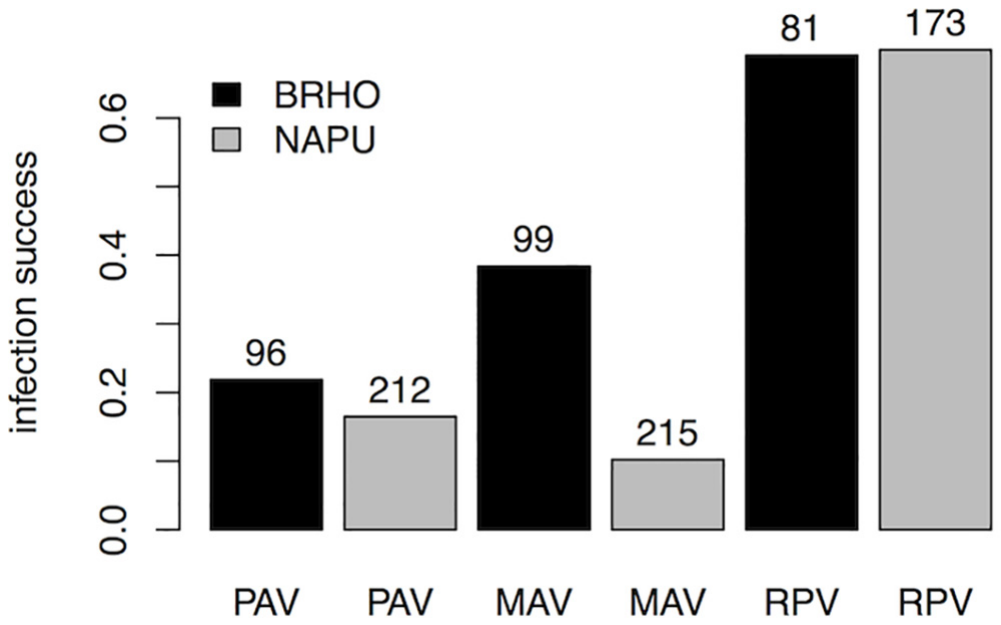
Fraction of inoculations that were successful for PAV, MAV, and RPV in *Bromus hordeaceus* (black bars) and *Nassella pulchra* (gray bars). Numbers above bars indicate sample sizes.

In the model that included all three viruses, inoculation success was not significantly lower in *Nassella* than in *Bromus* (p = 0.26) but MAV had lower success in *Nassella* (p = 0.0019; Table C in S1 Appendix; Fig 4). MAV and RPV had higher inoculation success than PAV (p = 0.013, p<0.001, respectively; Table C in S1 Appendix; Fig 4).

In summary, RPV, which had the highest inoculation success, was insensitive to the species identity of the host, MAV had lower inoculation success in *Nassella* (Fig 4). Although PAV and MAV coinfection was rare, there were no statistically significant effects of other viruses on infection success.

### Above-ground biomass

For the subset of plants for which treatments were fully successful, shoot biomass did not significantly differ between host species (p>0.7), but MAV infections had significantly lower shoot biomass (p = 0.028; Table D in S1 Appendix Model *m*_*0*_; Figure A in S1 Appendix, parts A-B). Only 21.2% of plants had fully successful treatments (i.e., acquired exactly the viruses with which they were inoculated; Table A in S1 Appendix). This low success rate was expected due to low transmission efficiency and interactions between viruses within co-inoculated plants (i.e., cross-protection; [16]), and it was the reason sample sizes were initially unequal across treatments.

**Fig 5:**
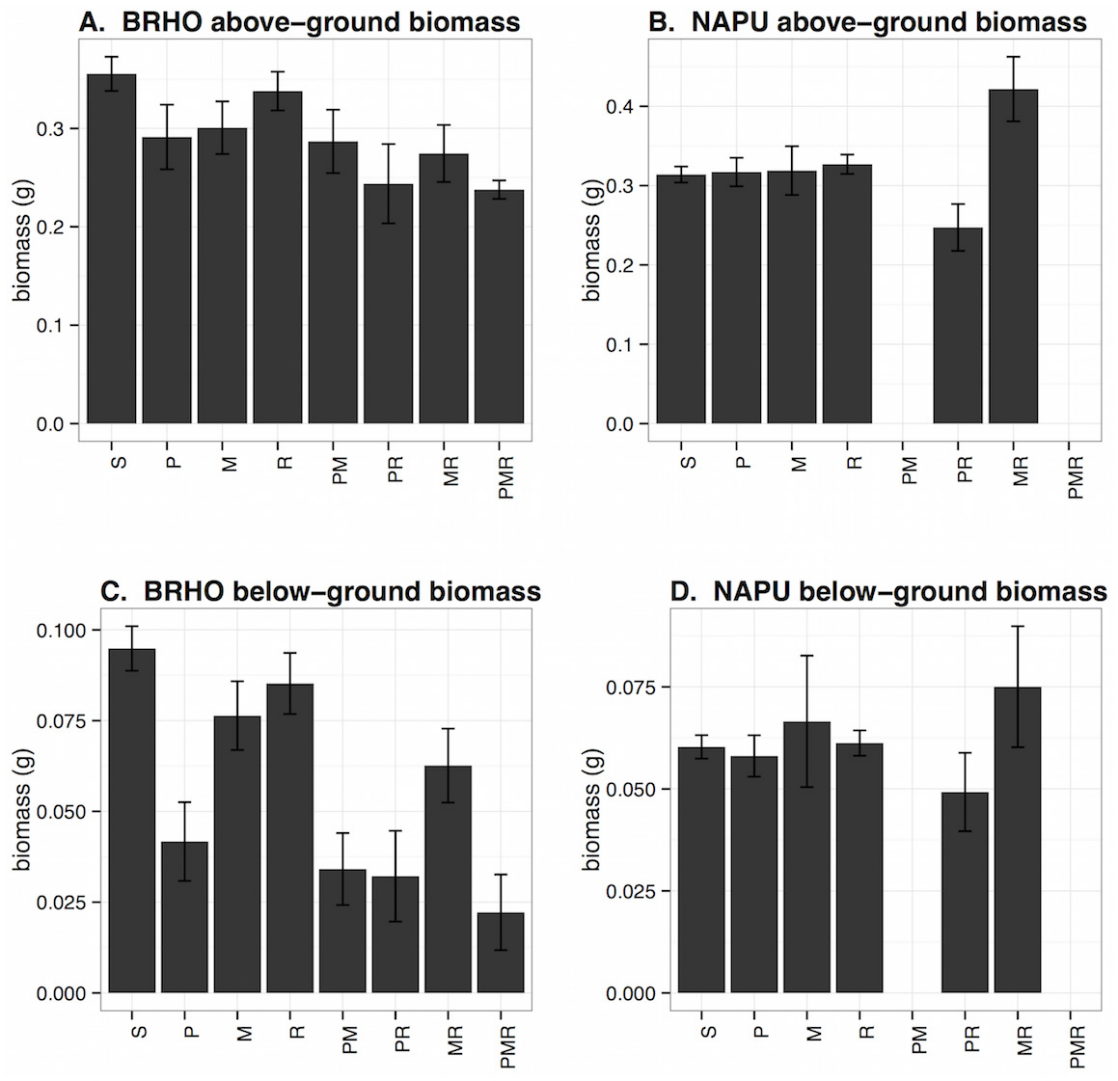
Biomass of plants in each infection class. A and B, above-ground biomass and C and D, below-ground biomass, both in grams. A and C are *Bromus hordeaceus* and B and D are *Nassella pulchra*. Infection classes are: S = susceptible, P = PAV only, M = MAV only, R = RPV only, PM = PAV-MAV, PR = PAV-RPV, MR = MAV-RPV, PMR = PAV-MAV-RPV. Data are from all plants in the experiment. Error bars are +/- 1 SE.

**Fig 6:**
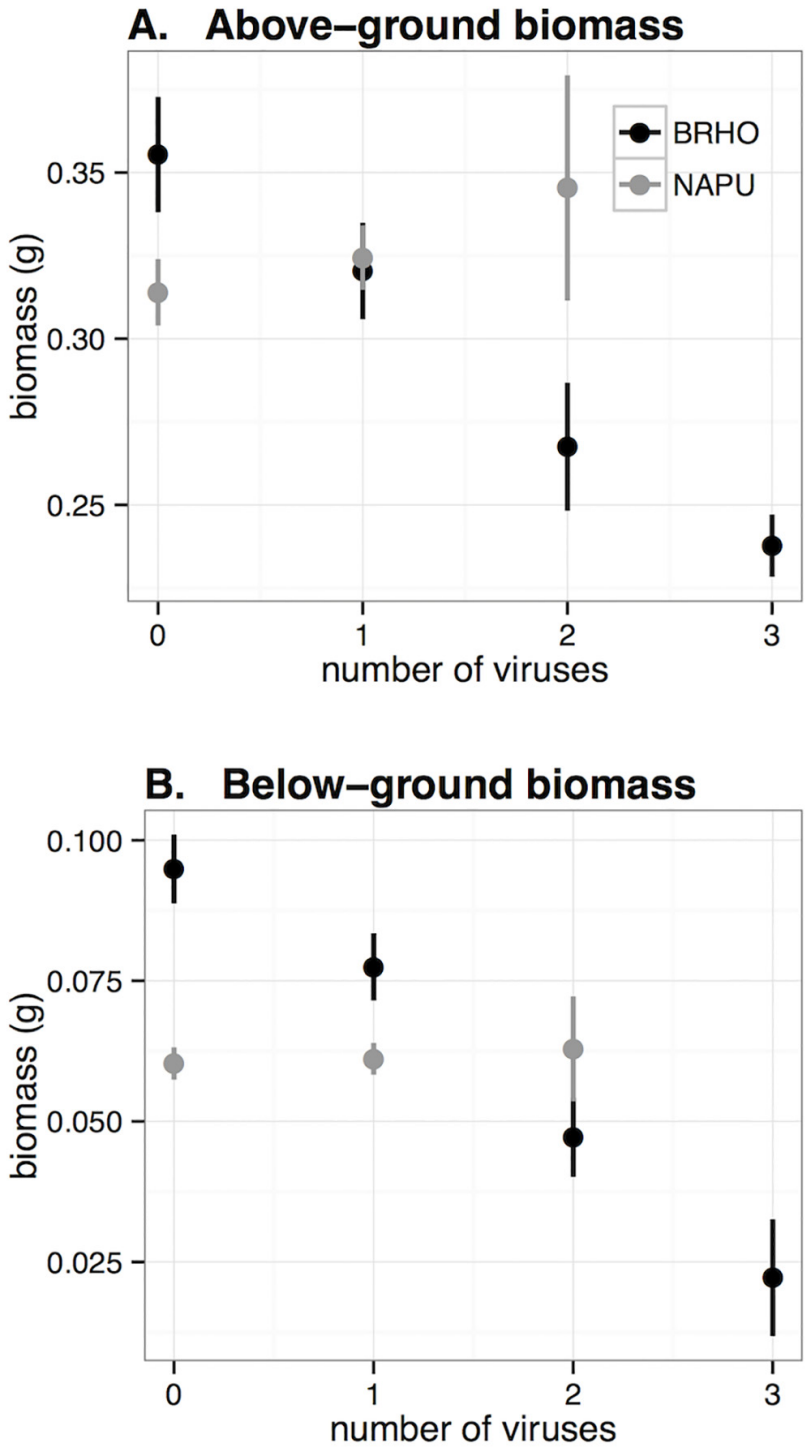
Above- and below-ground biomass versus number of viruses infecting a plant. A, above-ground biomass and B, below-ground biomass, both in grams. Black points are *Bromus hordeaceus* and gray points are *Nassella pulchra*. Data are from all plants in the experiment. Error bars are +/- 1 SE.

We then examined how plant species and infection affected shoot biomass across all plants, regardless of treatment success (Fig 5). The model that received the most support based on AIC was *m*_*7*_, which described shoot biomass as a function of species, number of viruses infection the plant, and their interaction (Table D in S1 Appendix). In that model, above-ground biomass of *Bromus* declined more strongly with the number of viruses infecting a plant than did above-ground biomass of *Nassella* (Fig 6A; Table D in S1 Appendix). In general, the models indicated that impacts of infection were greater on shoot biomass of *Bromus* than on *Nassella*.

### Below-ground biomass

For the subset of plants in which treatments were fully successful, *Nassella* had lower root biomass than *Bromus* (p<0.001). In addition, MAV, PAV-MAV, PAV-RPV, and PAV-MAV-RPV coinfections had significantly (p<0.02) lower root biomass than other infected and uninfected plants (Table E in S1 Appendix Model *m*_*0*_; Figure A in S1 Appendix, parts C-D).

Among the models of below-ground biomass that included all plants, the model that included plant species by virus number interactions was received the most support based on AIC (model *m*_*7*_; Table E in S1 Appendix). In that model, *Bromus* biomass declined more steeply than *Nassella* with infection by increasing numbers of viruses (p<0.001; Fig 6B). The next most-supported model (*m*_*6*_: Delta-AIC = 6) included plant species by infection status interactions. In that model, plants in all infection classes except RPV and MAV had significantly lower root biomass in *Bromus* (i.e., negative main effects; p<0.002), but not in *Nassella* (i.e., positive interactions; p<0.02; Table E in S1 Appendix; Fig 5C and 5D). In *Bromus*, plants infected or coinfected with PAV had notably lower root biomass than other plants (Fig 5C). This pattern was not apparent in *Nassella*, although two of the most potentially aggressive infection coinfections, PAV-MAV and PAV-MAV-RPV, were missing from this host species (Fig 5D). Roots responded similarly but more strongly to virus infection than shoots: for example, the correlation coefficient between parameter values in model *m*_*6*_ for above-ground and below-ground biomass was 0.91. In general, root and shoot biomass were highly correlated regardless of infection status, though shoot biomass began to saturate in plants with very high root biomass (Figure B in S1 Appendix).

## Discussion

Based on previous work with BYDVs, we predicted that (1) the viruses would have lower inoculation success in *Nassella*, (2) PAV and MAV would each have lower inoculation success when the other virus was present in a plant, (3) PAV would increase RPV inoculation success, and (4) both *Nassella* and *Bromus* would experience biomass reductions when infected that would increase with within-host virus diversity. Each of these predictions was partially, but not fully, supported. *Nassella* had lower susceptibility to MAV than *Bromus*, but host species did not significantly differ in susceptibility to PAV or RPV (prediction 1). Although the infection data qualitatively fit our predictions about the directional effects of virus interactions on inoculation success—that PAV and MAV would suppress each other and that PAV would facilitate RPV (predictions 2–3)—the effects were not statistically significant (Fig 3). Finally, *Bromus* above- and below-ground biomass decreased by approximately 0.04 and 0.02 grams per infecting virus, respectively, but within-host virus diversity did not strongly affect *Nassella* biomass (prediction 4).

Infection reduced biomass by up to 33% and 77% in *Bromus* and by up to 21% and 18% in *Nassella* above- and below-ground, respectively. Infected *Bromus* plants had lower above- and below-ground biomass than uninfected *Bromus*, and biomass declined with the number of viruses present; however, these patterns did not hold for *Nassella*, which had lower biomass than *Bromus* regardless of infection status but no significant effect of infection or virus number (Fig 6). In general, infections and coinfections that included PAV were most virulent, particularly in their effect on root biomass (Fig 5). By contrast, plants infected with MAV and/or RPV (but not PAV) showed little effect of infection on above- or below-ground biomass.

Because *Nassella* was less susceptible to virus infection and experienced smaller reductions in biomass when infected (suggesting higher tolerance for infection), we might expect viruses to play a relatively minor role for *Nassella* populations in the field. However, since *Nassella* is a perennial, any effects of infection may compound year after year by reducing biomass, survival, and fecundity in the long term [12,17]. As a result, even small reductions in over-summer survival could have major impacts on the ability of native perennial grasses like *Nassella* to coexist with exotic annual grasses like *Bromus* in California grasslands. Ideally, we would measure fitness effects on inoculated plants in the field, but such experiments are logistically challenging because they require access to local virus, aphid, and plant populations and control over aphid exposure throughout the growing season.

By measuring the effect of single- and multiple- infection on above- and below-ground biomass for two common plant species, this study highlights individual-level consequences of infection. A major future direction is to translate these individual effects to population- and community-level outcomes, using models parameterized based on the focal species. Pathogens that differentially affect the fitness of multiple host species can influence the outcome of competition between hosts [29], and previous work on BYDVs in California grasslands suggested that the virus could suppress native perennial grasses (like *Nassella*) and favor the invasion of exotic annual grasses (like *Bromus*) [12]. Yet it remains unclear how simultaneous infection by multiple pathogens affects this outcome, though coinfection is common in the field [8]. This study fills an important empirical gap in understanding the fitness impacts of coinfection. Future work should address the quantitative effects of these pathogens on population growth, and thereby on the community composition, of competing grass species.

With growing recognition that humans, plants, and wildlife host many interacting infectious agents, recent work has focused on understanding the factors controlling coinfection [30–32], and its consequences for transmission and disease outcomes for the host individual and population [33–36]. Studies in plants [8–10], (e.g., this study, [37]), mice [30,33], amphibians [31,36], and other experimentally tractable systems are critical for translating ecological processes well-understood in the free-living world—e.g., resource competition, apparent competition, dispersal, colonization—into disease systems, particularly those that are less experimentally tractable such as human diseases. Finally, our study demonstrates how pathogens can more substantially reduce host performance in coinfection. While an increasing richness of coinfecting pathogens can more severely decrease host performance, this effect of virus diversity can vary among host species.

## Supporting Information

S1 Appendix This appendix contains Tables A-E and Figs A-B. (PDF)

S1 File This file contains the biomass and infection data used in the study. (XLSX)
