# Supplementary material for: Differential Impacts of Virus Diversity on Biomass Production of a Native and an Exotic Grass Host
Source: PLoS One. 2015 Jul 31;10(7):e0134355. doi: 10.1371/journal.pone.0134355 (PMC4521826; doi:10.1371/journal.pone.0134355)
Supplement: S1 Appendix — (PDF) [file pone.0134355.s001.pdf]

***PLoS One* Supporting Information Appendix S1**

Article title: Differential impacts of virus diversity on biomass production of a native and an exotic grass host

Authors: Mordecai, E.A., Hindenlang, M., Mitchell, C.E.

The following Supporting Information is available for this article:

S1 Table A. Experiment sample sizes. Sample size in each inoculation treatment for *Bromus* and *Nassella* and the number of plants with each observed infection status in each treatment group.

S1 Table B. Inoculation success GLMs. Binomial GLMs of inoculation success for each virus as a function of plant species and infection status with respect to the other two viruses.

S1 Table C. Inoculation success GLMM. Binomial GLMM of inoculation success as a function of plant species and focal virus species.

S1 Table D. Above-ground biomass models. Linear models of above-ground biomass as a function of plant species and infection.

S1 Table E. Below-ground biomass models. Linear models of below-ground biomass as a function of plant species and infection.

S1 Fig. A. Biomass by successful inoculation treatment. Biomass of plants successfully inoculated with each treatment.

S1 Fig. B. Above-ground versus below-ground biomass. Above-ground versus below-ground biomass for each plant, plotted by species and infection status.

Table A. Sample size in each inoculation treatment for *Bromus* and *Nassella* and the number of plants with each observed infection status in each treatment group. Sample sizes for *Nassella* were initially double those for *Bromus* because of previous evidence of lower inoculation success in perennial grasses (e.g., Cronin et al. 2010). However, some samples were discarded due to an error during ELISA assays (see Methods). Treatments are: no aphids, controls with 5 unexposed *R. padi* (Con-5-Rp), controls with 5 unexposed *S. avenae* (Con-5-Sa), controls with 5 unexposed *R. padi* and 5 uninfected *S. avenae* (Con-5-Rp-5-Sa), 5 PAV-exposed *R. padi* (PAV), 5 MAV-exposed *S. avenae* (MAV), 5 RPV-exposed *R. padi* (RPV), 5 PAV-exposed *R. padi* and 5 MAV-exposed *S. avenae* (PAV+MAV), 5 PAV-RPV-double-exposed *R. padi* (PAV+RPV), 5 MAV-exposed *S. avenae* and 5 RPV-exposed *R. padi* (MAV+RPV), and 5 PAV-RPV-double-exposed *R. padi* and 5 MAV-exposed *S. avenae* (PAV+MAV+RPV). The number of fully successful inoculations for each treatment is shown in bold. Observed infection statuses are uninfected (i.e., susceptible; S), PAV-infected (P), MAV-infected (M), RPV-infected (R), PAV-MAV-coinfected (PM), PAV-RPV-coinfected (PR), MAV-RPV-coinfected (MR), and PAV-MAV-RPV-coinfected (PMR).

Table A

Treatment	Sample size	Observed infection status							
		S	P	M	R	PM	PR	MR	PMR
Bromus hordeaceus									
No treatment	3	**3**	0	0	0	0	0	0	0
Con-5-Rp	4	**4**	0	0	0	0	0	0	0
Con-5-Sa	5	**5**	0	0	0	0	0	0	0
Con-5-Rp-5-Sa	5	**5**	0	0	0	0	0	0	0
PAV	9	9	**0**	0	0	0	0	0	0
MAV	9	5	0	**4**	0	0	0	0	0
RPV	10	4	0	0	**6**	0	0	0	0
PAV + MAV	36	17	6	8	0	**5**	0	0	0
PAV + RPV	17	4	1	0	8	0	**4**	0	0
MAV + RPV	20	4	0	4	7	0	0	**5**	0
PAV + MAV + RPV	34	4	0	4	15	0	3	6	**2**
Nassella pulchra									
No treatment	2	**2**	0	0	0	0	0	0	0
Con-5-Rp	9	**9**	0	0	0	0	0	0	0
Con-5-Sa	8	**8**	0	0	0	0	0	0	0
Con-5-Rp-5-Sa	8	**8**	0	0	0	0	0	0	0
PAV	20	17	**3**	0	0	0	0	0	0
MAV	19	17	0	**2**	0	0	0	0	0
RPV	20	5	0	0	**15**	0	0	0	0
PAV + MAV	79	52	18	9	0	**0**	0	0	0
PAV + RPV	36	9	3	0	20	0	**4**	0	0
MAV + RPV	40	7	0	1	26	0	0	**6**	0
PAV + MAV + RPV	77	24	2	1	42	0	5	3	**0**
**Totals:**	**470**	**222**	**33**	**33**	**139**	**5**	**16**	**20**	**2**

Table B. Binomial GLMs of inoculation success for each virus as a function of plant species (NAPU = *Nassella pulchra*) and infection status with respect to the other two viruses. Coefficient estimates with p-values <0.05 are shown in bold (all values rounded to three decimal places). The baseline treatment in each model is *Bromus* uninfected with the other two viruses.

	Estimate	Standard Error	z-value	P-value
PAV status ~ Species + MAV status + RPV status				
**Intercept**	**-1.137**	**0.299**	**-3.799**	**0.000**
NAPU	-0.391	0.322	-1.214	0.225
MAV infected	-0.158	0.468	-0.337	0.736
RPV infected	-0.253	0.316	-0.800	0.424
MAV status ~ Species + PAV status + RPV status				
Intercept	-0.372	0.252	-1.474	0.140
**NAPU**	**-1.718**	**0.307**	**-5.594**	**0.000**
PAV infected	-0.363	0.469	-0.773	0.440
RPV infected	-0.119	0.317	-0.376	0.707
RPV status ~ Species + PAV status + MAV status				
**Intercept**	**0.778**	**0.272**	**2.857**	**0.004**
NAPU	0.046	0.305	0.150	0.881
PAV infected	0.296	0.494	0.599	0.549
MAV infected	-0.022	0.426	-0.053	0.958

Table C. Binomial GLMM of inoculation success as a function of plant species (NAPU = *Nassella pulchra*) and focal virus species. The baseline treatment is *Bromus* inoculated with PAV. Coefficient estimates with p-values <0.05 are shown in bold (all values rounded to three decimal places).

	Estimate	Standard Error	z value	P-value
**Intercept**	**-1.277**	**0.247**	**-5.161**	**0.000**
NAPU	-0.348	0.309	-1.125	0.261
**MAV**	**0.802**	**0.322**	**2.488**	**0.013**
**RPV**	**2.086**	**0.345**	**6.045**	**0.000**
**NAPU x MAV**	**-1.354**	**0.435**	**-3.114**	**0.002**
NAPU x RPV	0.386	0.426	0.908	0.364

Table D. Linear models of above-ground biomass as a function of plant species (NAPU = *Nassella pulchra*) and infection. Model m_0 uses data from only the plants in which the treatment was successful, whereas the remaining models use data from all plants (AIC shown for these models only). See Methods for model descriptions. P-values are rounded to the third decimal point, and coefficient estimates with p-values < 0.05 are shown in bold. The baseline treatment in all models is uninfected *Bromus* (Con-5-Rp *Bromus* in model m_0).

Table D

	Estimate	Std. Error	t-value	P-value	AIC
m_0: Biomass ~ Plant + Treatment					
**Intercept**	**0.395**	**0.038**	**10.269**	**0.000**	
NAPU	-0.009	0.027	-0.345	0.731	
Con-5-Rp-5-Sa	-0.056	0.048	-1.163	0.248	
Con-5-Sa	-0.011	0.047	-0.226	0.822	
**MAV**	**-0.141**	**0.063**	**-2.240**	**0.028**	
MAV + RPV	-0.024	0.050	-0.483	0.630	
No treatment	-0.030	0.069	-0.430	0.669	
PAV	-0.095	0.077	-1.239	0.219	
PAV + MAV	-0.108	0.065	-1.660	0.101	
PAV + MAV + RPV	-0.157	0.092	-1.715	0.090	
PAV + RPV	-0.105	0.056	-1.867	0.065	
RPV	-0.035	0.043	-0.823	0.413	
m_1: Biomass ~ Plant					***-622***
**Intercept**	**0.326**	**0.010**	**32.528**	**0.000**	
NAPU	-0.006	0.012	-0.502	0.616	
m_2: Biomass ~ PAV status + MAV status + RPV status					***-623***
**Intercept**	**0.328**	**0.008**	**41.812**	**0.000**	
**PAV status**	**-0.037**	**0.018**	**-2.118**	**0.035**	
MAV status	-0.008	0.017	-0.442	0.659	
RPV status	0.000	0.012	0.010	0.992	
m_3: Biomass ~ Infection class					***-619***
**Intercept**	**0.326**	**0.008**	**39.647**	**0.000**	
M	-0.018	0.023	-0.785	0.433	
MR	0.018	0.029	0.628	0.530	

P	-0.014	0.023	-0.636	0.525
PM	-0.039	0.055	-0.719	0.473
PMR	-0.088	0.086	-1.029	0.304
**PR**	**-0.081**	**0.033**	**-2.417**	**0.016**
R	0.004	0.013	0.276	0.783
m_4: Biomass ~ Plant x [PAV status + MAV status + RPV status]				***-625***
**Intercept**	**0.356**	**0.014**	**25.300**	**0.000**
**NAPU**	**-0.042**	**0.017**	**-2.476**	**0.014**
**PAV status**	**-0.060**	**0.028**	**-2.101**	**0.036**
**MAV status**	**-0.048**	**0.023**	**-2.064**	**0.040**
RPV status	-0.025	0.021	-1.211	0.226
NAPU x PAV	0.045	0.036	1.256	0.210
**NAPU x MAV**	**0.089**	**0.035**	**2.531**	**0.012**
NAPU x RPV	0.039	0.025	1.528	0.127
m_5: Biomass ~ Plant + Infection class				***-618***
**Intercept**	**0.335**	**0.012**	**27.493**	**0.000**
NAPU	-0.012	0.013	-0.978	0.328
M	-0.022	0.023	-0.936	0.350
MR	0.015	0.029	0.525	0.600
P	-0.013	0.023	-0.592	0.554
PM	-0.048	0.055	-0.868	0.386
PMR	-0.097	0.086	-1.125	0.261
**PR**	**-0.083**	**0.033**	**-2.486**	**0.013**
R	0.004	0.013	0.314	0.754
m_6: Biomass ~ Plant x Infection class				***-620***
**Intercept**	**0.355**	**0.015**	**23.526**	**0.000**
**NAPU**	**-0.041**	**0.018**	**-2.310**	**0.021**

M	-0.055	0.031	-1.744	0.082	
**MR**	**-0.081**	**0.041**	**-1.982**	**0.048**	
P	-0.064	0.048	-1.344	0.180	
PM	-0.069	0.056	-1.233	0.218	
PMR	-0.118	0.086	-1.366	0.173	
**PR**	**-0.112**	**0.048**	**-2.337**	**0.020**	
R	-0.018	0.026	-0.679	0.497	
NAPU x M	0.060	0.047	1.276	0.202	
**NAPU x MR**	**0.189**	**0.058**	**3.255**	**0.001**	
NAPU x P	0.067	0.054	1.246	0.214	
NAPU x PR	0.045	0.067	0.675	0.500	
NAPU x R	0.031	0.030	1.016	0.310	
m_7: Biomass ~ Plant + Number of viruses					***-629***
**Intercept**	**0.357**	**0.014**	**25.619**	**0.000**	
**NAPU**	**-0.044**	**0.017**	**-2.629**	**0.009**	
**number of viruses**	**-0.042**	**0.013**	**-3.169**	**0.002**	
**NAPU x number of viruses**	**0.054**	**0.017**	**3.104**	**0.002**	
m_8: Biomass ~ Plant x Infection status					***-625***
**Intercept**	**0.355**	**0.015**	**23.402**	**0.000**	
**NAPU**	**-0.041**	**0.018**	**-2.297**	**0.022**	
**Infected**	**-0.051**	**0.020**	**-2.534**	**0.012**	
**NAPU x Infected**	**0.064**	**0.024**	**2.609**	**0.009**	

Table E. Linear models of below-ground biomass as a function of plant species and infection. Model m_0 uses data from only the plants in which the treatment was successful, whereas the remaining models use data from all plants (AIC shown for these models only). See Methods for model descriptions. P-values are rounded to the third decimal point, and coefficient estimates with p-values < 0.05 are shown in bold. The baseline treatment in all models is uninfected *Bromus* (Con-5-Rp *Bromus* in model m_0).

Table E

	Estimate	Std. Error	t-value	P-value	AIC
m_0: Biomass ~ Plant + Treatment					
**Intercept**	**0.108**	**0.012**	**9.336**	**0.000**	
**NAPU**	**-0.037**	**0.008**	**-4.634**	**0.000**	
Con-5-Rp-5-Sa	-0.016	0.015	-1.121	0.266	
Con-5-Sa	0.008	0.014	0.593	0.555	
**MAV**	**-0.039**	**0.019**	**-2.049**	**0.044**	
MAV + RPV	-0.029	0.015	-1.909	0.060	
No	-0.015	0.019	-0.792	0.430	
PAV	-0.034	0.023	-1.457	0.149	
**PAV + MAV**	**-0.074**	**0.020**	**-3.690**	**0.000**	
**PAV + MAV + RPV**	**-0.085**	**0.028**	**-3.034**	**0.003**	
**PAV + RPV**	**-0.040**	**0.016**	**-2.435**	**0.017**	
RPV	-0.004	0.013	-0.285	0.777	
m_1: Biomass ~ Plant					***-1666***
**Intercept**	**0.080**	**0.003**	**24.421**	**0.000**	
**NAPU**	**-0.019**	**0.004**	**-4.725**	**0.000**	
m_2: Biomass ~ PAV status + MAV status + RPV status					***-1655***
**Intercept**	**0.071**	**0.003**	**27.564**	**0.000**	
**PAV status**	**-0.022**	**0.006**	**-3.762**	**0.000**	
MAV status	-0.001	0.006	-0.172	0.863	
RPV status	-0.004	0.004	-1.057	0.291	
m_3: Biomass ~ Infection class					***-1649***
**Intercept**	**0.070**	**0.003**	**25.904**	**0.000**	
M	0.002	0.008	0.271	0.786	
MR	-0.002	0.009	-0.223	0.824	

**P**	**-0.016**	**0.008**	**-2.086**	**0.038**	
**PM**	**-0.036**	**0.018**	**-1.983**	**0.048**	
PMR	-0.048	0.029	-1.678	0.094	
**PR**	**-0.029**	**0.011**	**-2.698**	**0.007**	
R	-0.003	0.004	-0.649	0.516	
m₄: Biomass ~ Plant + PAV status + MAV status + RPV status					***-1696***
**Intercept**	**0.095**	**0.004**	**21.302**	**0.000**	
**NAPU**	**-0.034**	**0.005**	**-6.442**	**0.000**	
**PAV status**	**-0.049**	**0.009**	**-5.441**	**0.000**	
**MAV status**	**-0.018**	**0.007**	**-2.478**	**0.014**	
RPV status	-0.011	0.007	-1.669	0.096	
**NAPU x PAV**	**0.045**	**0.011**	**3.902**	**0.000**	
**NAPU x MAV**	**0.027**	**0.011**	**2.444**	**0.015**	
NAPU x RPV	0.012	0.008	1.456	0.146	
m_5: Biomass ~ Plant + Infection class					***-1676***
**Intercept**	**0.086**	**0.004**	**22.035**	**0.000**	
**NAPU**	**-0.022**	**0.004**	**-5.437**	**0.000**	
M	-0.005	0.008	-0.617	0.537	
MR	-0.008	0.009	-0.852	0.395	
P	-0.014	0.007	-1.915	0.056	
**PM**	**-0.052**	**0.018**	**-2.890**	**0.004**	
**PMR**	**-0.064**	**0.028**	**-2.281**	**0.023**	
**PR**	**-0.033**	**0.010**	**-3.148**	**0.002**	
R	-0.002	0.004	-0.513	0.608	
m_6: Biomass ~ Plant x Infection class					***-1685***
**Intercept**	**0.095**	**0.005**	**19.689**	**0.000**	
**NAPU**	**-0.035**	**0.006**	**-6.050**	**0.000**	
M	-0.019	0.010	-1.837	0.067	

**MR**	**-0.032**	**0.013**	**-2.563**	**0.011**	
**P**	**-0.053**	**0.015**	**-3.465**	**0.001**	
**PM**	**-0.061**	**0.018**	**-3.394**	**0.001**	
**PMR**	**-0.073**	**0.028**	**-2.625**	**0.009**	
**PR**	**-0.063**	**0.015**	**-4.087**	**0.000**	
R	-0.010	0.008	-1.192	0.234	
NAPU x M	0.025	0.015	1.649	0.100	
**NAPU x MR**	**0.047**	**0.018**	**2.575**	**0.010**	
**NAPU x P**	**0.051**	**0.017**	**2.931**	**0.004**	
**NAPU x PR**	**0.052**	**0.021**	**2.490**	**0.013**	
NAPU x R	0.011	0.009	1.116	0.265	
m_7: Biomass ~ Plant + Number of viruses					***-1691***
**Intercept**	**0.097**	**0.004**	**21.668**	**0.000**	
**NAPU**	**-0.036**	**0.005**	**-6.791**	**0.000**	
**number of viruses**	**-0.023**	**0.004**	**-5.453**	**0.000**	
**NAPU x number of viruses**	**0.024**	**0.006**	**4.285**	**0.000**	
m_8: Biomass ~ Plant x Infection status					***-1679***
**Intercept**	**0.095**	**0.005**	**19.345**	**0.000**	
**NAPU**	**-0.035**	**0.006**	**-5.944**	**0.000**	
**Infected**	**-0.027**	**0.006**	**-4.138**	**0.000**	
**NAPU x Infected**	**0.028**	**0.008**	**3.545**	**0.000**	

Supplementary Figures

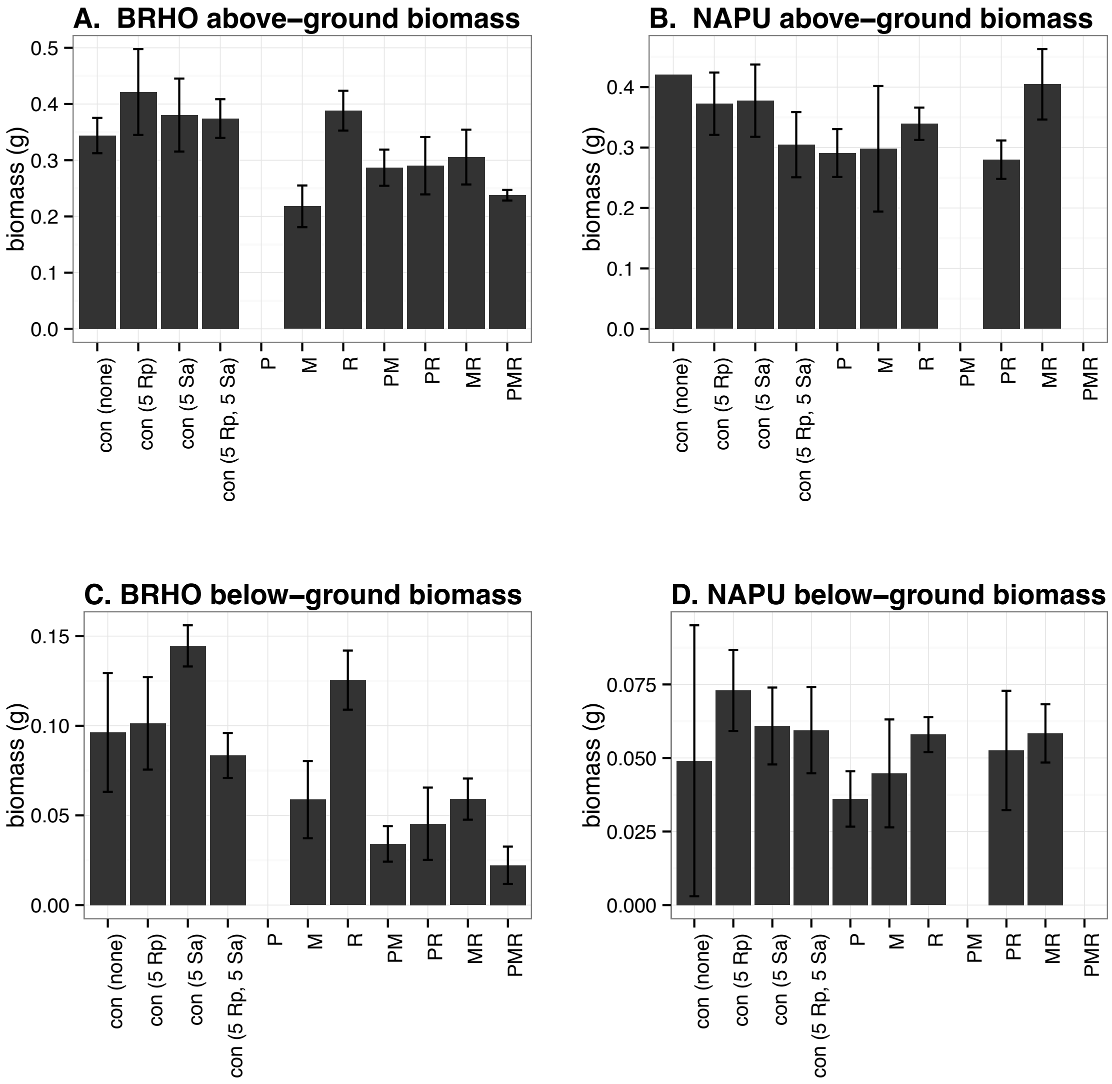

Fig. A. Biomass of plants successfully inoculated with each treatment. A and B, above-ground biomass and C and D, below-ground biomass, both in grams. A and C are *Bromus hordeaceus* and B and D are *Nassella pulchra*. The control treatments are: con (none) = no aphids, con (5 Rp) = 5 uninfected *R. padi* aphids, con (5 Sa) = 5 uninfected *S. avenae* aphids, and con (5 Rp, 5 Sa) = 5 uninfected *R. padi* and 5 uninfected *S. avenae* aphids. The infected treatments are: P = PAV only, M = MAV only, R = RPV only, PM = PAV-

MAV, PR = PAV-RPV, MR = MAV-RPV, PMR = PAV-MAV-RPV. None of the *Bromus* PAV-only or the *Nassella* PAV-MAV or PAV-MAV-RPV treatments were successful. Error bars are +/- 1 SE.

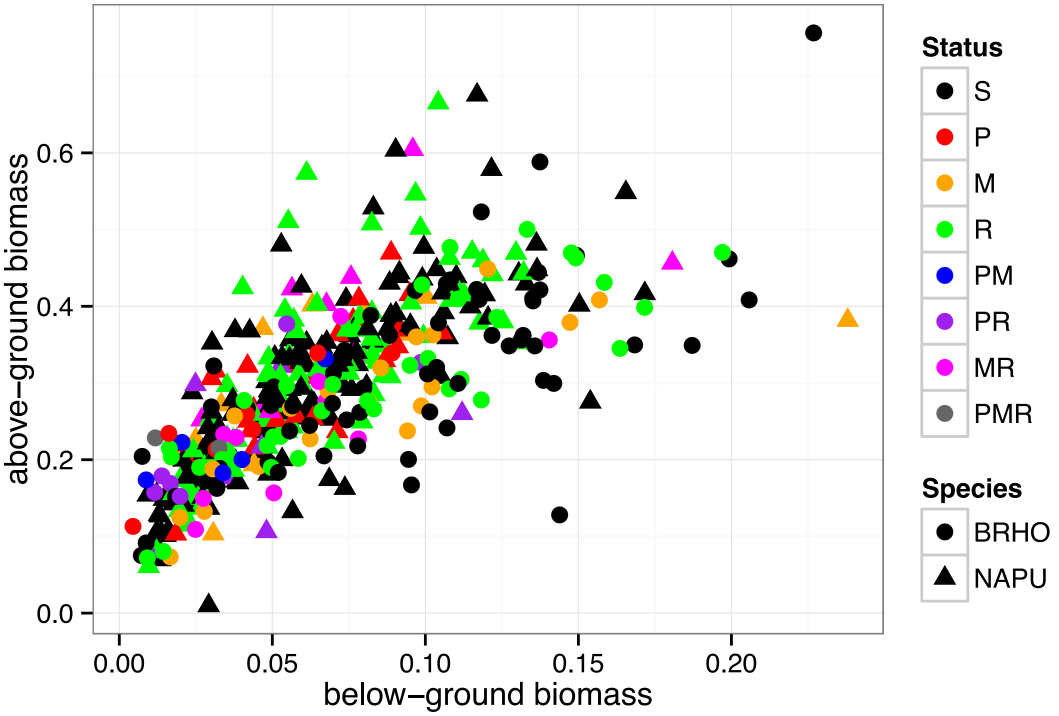

Fig. B. Above-ground versus below-ground biomass for each plant, plotted by species and infection status. Points are colored based on infection status, which use the same codes as in Fig. 5. Circles are *Bromus hordeaceus* and triangles are *Nassella pulchra*. Biomass values are in grams.
